# Prevalence and characteristics of referred pain in patients diagnosed with temporomandibular disorders according to the Diagnostic Criteria for Temporomandibular Disorders (DC/TMD)

**DOI:** 10.12688/f1000research.109696.1

**Published:** 2022-06-14

**Authors:** Nawal Alketbi, Wael Talaat

**Affiliations:** 1 Oral and Craniofacial Health Sciences, University of Sharjah, Sharjah, 27272, United Arab Emirates; 2 Research Institute for Medical and Health Sciences, University of Sharjah, Sharjah, 27272, United Arab Emirates; 3 Oral and Maxillofacial Surgery, Suez Canal University, Ismaillia, Egypt

**Keywords:** Temporomandibular disorders, Temporomandibular joint, Referred pain, Orofacial pain, Diagnostic Criteria for Temporomandibular Disorders

## Abstract

Background: Referred pain often complicates and delays the diagnosis of temporomandibular disorders (TMD). Elaborating the prevalence and characteristics of TMD-associated referred pain as well as the distribution of referred pain in different TMD classes will significantly improve the diagnostic process. The objectives of the present study were to assess the prevalence and to evaluate the characteristics of referred pain associated with TMD diagnosed according to the DC/TMD.

Methods: A total of 252 patients were evaluated using the DC/TMD Axes-I and –II assessment tools. Different modalities were used to treat the diagnosed TMD. Referred pain was diagnosed when the location of the perceived pain in response to palpation extended beyond the boundary of the structure that was examined. For pain locations that were perceived as deep, patients were asked to locate the surface of the area of pain. The result of the assessment was identified as positive if the patient described his perceived pain during the clinical examination as being familiar pain that was experienced in the same location in the last 30 days.

Results: TMD-associated referred pain was recorded in 153 patients (60.7%). The most common referred pain location was the temporal area (45.2%), followed by the ear (42.1%). The referred pain was recorded in disc displacement with reduction with intermittent locking and myofascial pain with referral in all patients (100%). The proportion of patients with referred pain was significantly different between the different TMD diagnostic subgroups (P < 0.001).  The recorded percentage of improvement in the referred pain following the treatment was 50.41% after 3 months and 56.65% after 6 months.

Conclusions: Referred pain is a prominent feature of TMD. More studies are required with longer follow-up periods and bigger sample sizes to support the findings of the present study.

## Introduction

Temporomandibular disorders are a combination of muscular, skeletal, and neuromuscular pathologic conditions that influence the temporomandibular joint (TMJ), the muscles of mastication, and the related tissues. The common symptoms of TMD are headache (occipital area, temporal area, and forehead), pain during chewing food, pain on opening or closing the jaws, TMJ pain, pain in the back of the neck, joint sounds, deviation upon opening the mouth, and limitation of mouth opening. ^1^ The pain associated with TMD is often a concatenation of diverse entangled pain sources. Microtrauma due to bruxism, joint laxity, and functional overloading are reported as the main causes of TMD, whereas TMD have been described as the most prevalent cause of non-odontogenic orofacial pain. ^2^ The reported prevalence of TMD was contradicting among studies, mainly due to the different evaluation techniques, diagnostic criteria and different TMD taxonomy used in different studies. ^3^^–^^9^ Patients tend to ignore their TMD symptoms which can lead to more advanced forms of these disorders. The prevalence of incidentally discovered TMD was 10.8% in one study, among whom osteoarthritis was diagnosed in 11.69%. ^10^

The diagnosis of TMD is challenging even to the most experienced practitioner mainly due to the concurrence of symptoms among different disorders. TMD diagnosis is usually achieved through history, physical examination and different imaging modalities. The Research Diagnostic Criteria for Temporomandibular Disorders (RDC/TMD) is a reliable instrument for TMD diagnosis, and it incorporates two axes; Axis-I involves the clinical and the radiographical diagnostic criteria, whereas Axis-II focuses on the evaluation of the psychosocial status and pain-related disabilities. ^11^ An updated diagnostic protocol that relied on strong evidence was published in 2014 to enhance the sensitivity and specificity of the original RDC/TMD. The modified protocol was identified as the Diagnostic Criteria for Temporomandibular Disorders (DC/TMD), and included new Axis-I assessment algorithms, and new Axis-II evaluation methods. The DC/TMD instruments proposed new nomenclature for different TMD classes, as it expanded to evolve new instruments on evaluating pain perspective, psychological standing and psychosocial bahavior. ^12^

TMD referred pain has been described as a familiar pain that spreads to an anatomically distant location away from the boundaries of the muscle or joint being examined. Familiar pain is the pain that can be reproduced in response to clinical provocation and has been experienced by the patient in the same anatomical location for 30 days prior to the clinical setting. ^12^ It is often challenging to the patient to identify the location of referred pain that spreads to deeper structures. Referred pain often complicates the diagnosis of TMD, as pain appears to originate from areas outside the anatomical boundaries of the joint and the associated muscles. The information about the TMD referred pain is very limited in the literature. Elaborating the prevalence and characteristics of referred pain associated with TMD will benefit the patients’ community as well as the medical professionals by facilitating the complex diagnostic process of these disorders.

The aim of this study was to assess the prevalence of referred pain associated with TMD diagnosed according to the DC/TMD, to evaluate the characteristics of TMD referred pain, and investigate the correlations between the distribution of referred pain and the different TMD classes.

## Methods

The study was performed at the College of Dental Medicine, University of Sharjah, United Arab Emirates, between February 2020 to June 2021. Ethical approval was granted by the Institutional Review Board at the University of Sharjah (REC-20-11-02-01-S). The ethical principles for medical research involving human subjects as mentioned in the Declaration of Helsinki were applied in this study. Comprehensive TMJ clinical examination was performed for all patients during their preliminary screening procedures. All patients who were initially diagnosed as having signs and symptom of TMD were enlightened about the objectives of the study and were asked to sign consent forms before participation.

All subjects aged 18 to 60 years with signs and symptoms of TMD that were diagnosed during the initial examination were invited to participate in this study. The signs and symptoms were clicking or crepitus sounds, TMJ pain, deviation in the path of mouth opening, limitation in mouth opening, headaches, ear pain and tenderness in the muscles of mastication. Patients were excluded from the study if they have been previously treated from TMD. Pregnant women were not enrolled in the study to prevent any detrimental effects from radiation. Patients were further evaluated using the DC/TMD assessment tools that included questionnaires, clinical examination, and radiographic examination. ^12^ Helkimos clinical dysfunction index (Di) was utilized to identify patients with severe dysfunction symptoms (group Di III) and refer them for cone beam computed tomography (CBCT) examination. The diagnosis of disc displacement was endorsed by the use of magnetic resonance imaging.

### DC/TMD questionnaires

The Axis-I assessment instruments used to evaluate all patients were the TMD Pain Screener, ^13^ Symptom Questionnaire ^14^ and Demographic Questionnaire. ^14^

### DC/TMD Axis I evaluation

Axis I-based clinical and radiographic examinations and structured diagnosis were carried out and included palpating the muscles of mastication and cervical muscles to diagnose the sites of tenderness or sustained contraction, auscultating, and palpating the joints during mandibular motion, observing the pattern of opening, and measuring the maximal mouth opening (pain free opening, maximum unassisted and assisted openings). Lateral excursions and the range of mandibular protrusion were also recorded. Pain level was assessed using the 10-point visual analogue scale (VAS) (0 indicating no pain and 10 indicating the most severe pain). The TMJ clinical evaluation was carried out by an oral and maxillofacial surgeon, who had over 15 years specialization in the management of TMDs. This examiner was blinded to the outcome of the initial screening procedure (performed by a general dental practitioner) as well as to any related outcomes that were evaluated in all questionnaires. Calibration of the examiners was accomplished before the start of the study by asking each examiner to assess the same 20 subjects independently. The results were then analyzed, and any disagreements were resolved through group discussion. Radiographic evaluation was done by using CBCT for any suspected degenerative joint diseases, whereas MRI was conducted to evaluate suspected internal derangement. All CBCT assessments were performed using GALILEOS 3-D X-ray systems (SIRONA Dental Systems, York, PA), whereas MRI examinations were performed using a 1.5 Tesla machine (CHORUS 1.5 T, ISOL Technology, Gyeonggi-do, South Korea) with a TMJ surface coil. The joints were evaluated observing any disc displacement with any indication of recapture. MRI and CBCT records were analyzed twice, with a 1-week interval, by a specialist in oral radiology with 32 years of experience. The radiologist was blinded to the clinical evaluation outcomes. TMD were then diagnosed and classified according to the DC/TMD Axis I criteria.

### DC/TMD Axis II evaluation

Pain drawings, Graded Chronic Pain Scale (GCPG), disability score, muscle tenderness score and Jaw Functional Limitations Scale were used as assessment tools for DC/TMD Axis II evaluation. A diagram was given for the patients to illustrate and shade the area of pain and referred pain. GCPG was measured with a time frame of 30 days and classified by using the 10-point VAS. The disability scale assessed the pain related dysfunction while opening the mouth, while eating and after waking up, and was graded as no disability = 0 points, mild disability = 1 point, moderate disability = 2 points and severe disability = 3 points. The muscle tenderness assessments was graded as no tenderness = 0 points, mild tenderness = 1 point, moderate tenderness = 2 points and severe tenderness = 3 points. Functional Limitations Scale was measured by asking the patients if they develop any limitation during chewing hard food, wide opening, eating, yawning and talking. Back pain, neck pain, ear pain, dizziness and trouble sleeping were also evaluated. All patients were asked about their oral behavior in regard to clenching or grinding teeth while sleeping or when awake.

### Treatment modalities for different TMD classes

**Conservative therapy**

Conservative therapy involved the use of stabilizing occlusal splints, soft diet, hot fomentation, limitation of mouth opening and non-steroidal anti-inflammatory drugs when needed. All patients were reevaluated postoperatively at 1, 3, and 6 months later to decide if any further treatment was required.

**Prolotherapy**

Prolotherapy was used for the treatment of patients with subluxation. The auriculo-temporal nerve was anaesthetized then 10% dextrose solution was injected at four locations. The first injection point was employed for the upper joint compartment, and the pericapsular tissues where the needle was inserted 10 mm anterior to the tragus and 2 mm below the canthal-tragus line. The needle was then directed anteromedially and 0.8 ml of the solution was injected intraarticularly. The needle was then withdrawn and 0.4 ml solution was injected into the pericapsular tissues. The second injection point was marked 2 mm below the canthal-tragus line and 1 cm in front of the mid-tragus and the needle was inserted at a depth of 3 mm from the skin surface targeting the superior capsular attachment and 0.4 mL of solution was deposited. Then the inferior capsular attachment was injected at the third injection point; 1 cm below the first one, and 0.4 mL of solution was applied. The forth injection point was placed anterior to the tragus and the needle was inserted to a depth of 20 mm to reach the posterior disc attachment and 0.4 mL of the solution was then applied. ^15^

**Arthrocentesis**

The skin was prepared with povidone iodine then local anesthetic solution was injected to block the auriculo-temporal nerve. For the double-needle arthrocentesis technique; the location for the first needle puncture point was decided by marking the canthal-tragus line and marking the puncture point at either 10 mm or 7 mm anterior to the tragus depending on the canthal-tragus distance, and 2 mm under the canthal-tragus line. The second needle was inserted at a point 10 mm anterior to the first point and 10 mm under the line. ^16^ The condyle was located and distracted downward and forward to widen the joint compartment and two 18-gage needles were inserted into each entry point. Single-needle arthrocentesis was done using a Y-shaped double-port cannula (Shepard cannula, Normed, Tuttlingen, Germany). The location for insertion was identical to the first needle in the double-needle technique. The joints were irrigated with 300 mL normal saline followed by the articular injection of 1 mL of low-molecular-weight Hyaluronic Acid (HA) (Hyalgan ^®^; Fidia, Abano Terme, Italy). TMJ physical exercise regimen started after the procedure and continued for one month. Splint therapy was provided, and all patients were reevaluated at 1 week, 1 mouth, 3 months, and 6 months.

**Botulinum toxin (Botox) injection**

Patients diagnosed with local myalgia and local myalgia with referral were treated by the injection of OnabotulinumtoxinA (BOTOX ^®^, Allergan Inc., Dublin, Ireland) combined with conservative treatment. Trigger points in the masseter muscle were injected by a maximum of 35 units, whereas the trigger points in the temporalis muscle were injected by a maximum of 25 units. The patients were recalled for follow up after one week and then at 1 mouth, 3 mouths and 6 months. The injections were repeated after 3 months if needed.

**Evaluation of referred pain associated with TMD**

Referred pain was confirmed as being positive or negative using a bilateral manual palpation technique. Palpation was done at the following sites; temporalis muscle, masseter muscle, lateral pole of the TMJ, around lateral pole of the TMJ, posterior mandible, submandibular area, lateral pterygoid muscle area and tendon of the temporalis. The patients were asked to bite slightly and relax to ensure palpation of the correct muscle site. The result of the assessment was identified as positive if the site of the perceived pain in response to palpation had extended past the outline of the structure that was examined. For pain locations that were perceived as deep, patients were asked to locate the surface of the area of pain. In addition, the result of the assessment was identified as positive only if the patient described his perceived pain during the clinical examination as being familiar pain that was experienced in the same location in the last 30 days before the examination. The correlations between the presence of referred pain and the TMJ pain level, duration of TMJ pain, maximal mouth opening, muscle tenderness and disability scores were assessed. The evaluation of referred pain was repeated following the treatment in each follow up session. The location of referred pain was recorded for each patient as well as the pain characteristics that were described as dull, sharp and throbbing and heterogeneous pain (mixed intermittent dull and sharp throbbing).

### Statistical analysis

Statistical analysis was performed using SPSS (version 20). Quantitative variables were described by the Mean, Standard Deviation (SD), Range (Minimum-Maximum), Standard Error (SE), and 95% confidence interval of the mean. Shapiro-Wilk test of normality was used to test the normality hypothesis of quantitative variables for further choice of appropriate parametric and non-parametric tests. Paired sample t-test was used for comparing pain levels at two-time points. Mann-Whitney U test was used for comparing mouth opening and duration between two groups. Qualitative categorical variables were described by proportions and Percentages. The χ ^2^ test of independence was used to determine the association between the 2 categorical variables. Various measures of correlation were applied as appropriate including; Cramer’s V measure of association, Point-biserial correlation coefficient, Rank biserial correlation coefficient and Phi correlation coefficient. Pareto Analysis was applied followed by the application of a z-test of 2 proportions to detect any statistically significant differences between the proportions of two groups. The significance level was set at P ˂0.05. Two-Tailed tests were assumed throughout the analysis for all statistical tests.

## Results

In the present study, 252 patients (137 male and 115 female) with a mean age of 34.19 ± 10.85 were enrolled. TMD were diagnosed in the left TMJ in 180 patients (71.4%), whereas the right TMJ was involved in 167 patients (66.3%). TMJ pain on one side was reported in 141 patients (56.0%), whereas bilateral TMJ pain was reported in 103 patients (40.9%). Anterior disc displacement with reduction was the most prevalent diagnosis (40.5%), followed by local myalgia (13.9%), anterior disc displacement without reduction with limited mouth opening (13.5%), myofascial pain with referral (11.5%), osteoarthritis (8.3%), hypermobility (6.0%), anterior disc displacement without reduction without limited mouth opening (5.2%) and disc displacement with reduction with intermittent locking (1.2%). Muscle tenderness was recorded on the masseter muscle in 76 patients (30.2%), in the temporalis muscle in 62 patients (24.6%) and in the lateral pterygoid muscle in 23 patients (9.1%).

**Figure 1.**
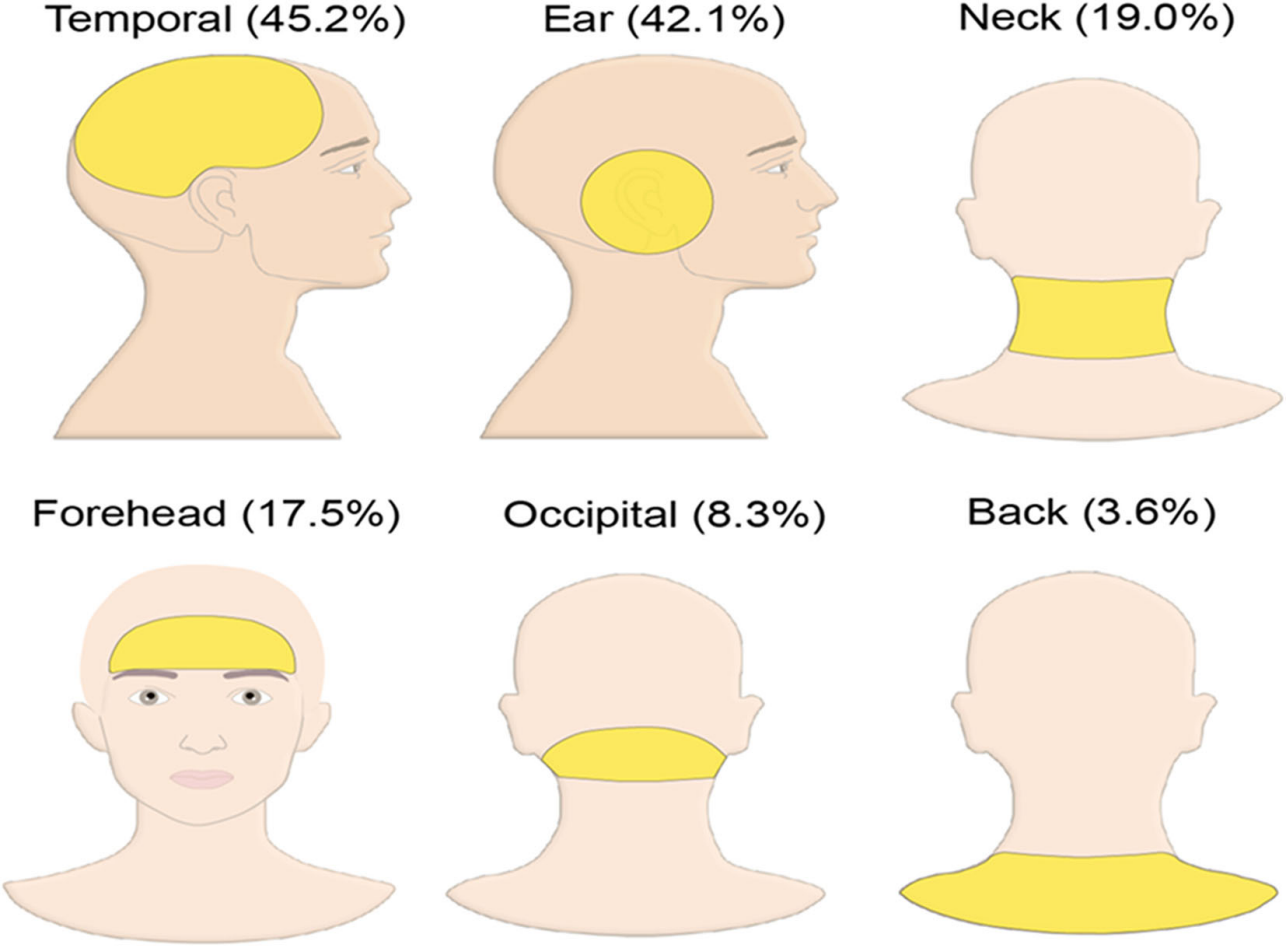
Prevalence of referred pain at different locations.

Referred pain was recorded in 153 patients (60.7%). The highest prevalence of referred pain was seen in the temporal area (45.2%), followed by the ear (42.1%), neck (19.0%) and forehead (17.5%) ( Figure 1). The prevalence of referred pain felt at more than one location in the same patient was 46.43% (117 patients). Referred pain has been recorded at only one location in 36 patients (14.3%), two locations in 70 patients (27.8%) and three locations in 27 patients (10.7%). The highest prevalence of referred pain reported at two locations was at temporal and ear (32.9%), followed by temporal and forehead (12.7%) then ear and neck (11.5%). The most frequent pain characteristic reported in the current study was dull type pain in 107 patients (42.5%), followed by sharp and throbbing pain in 91 patients (36.1%) and the heterogeneous pain in 6 patients (2.4%).

**Figure 2.**
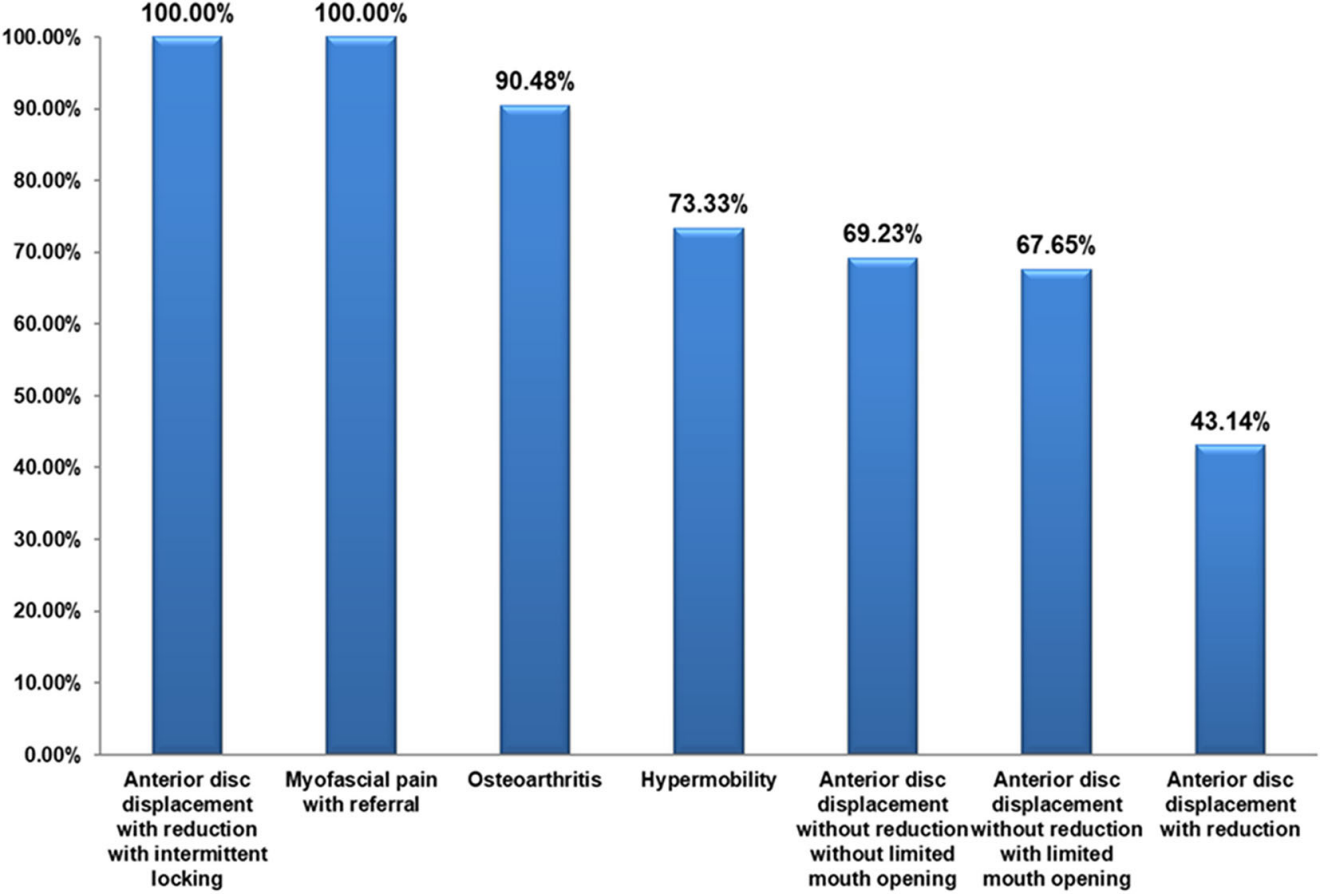
Prevalence of referred pain in different DC/TMD classes.

**Figure 3.**
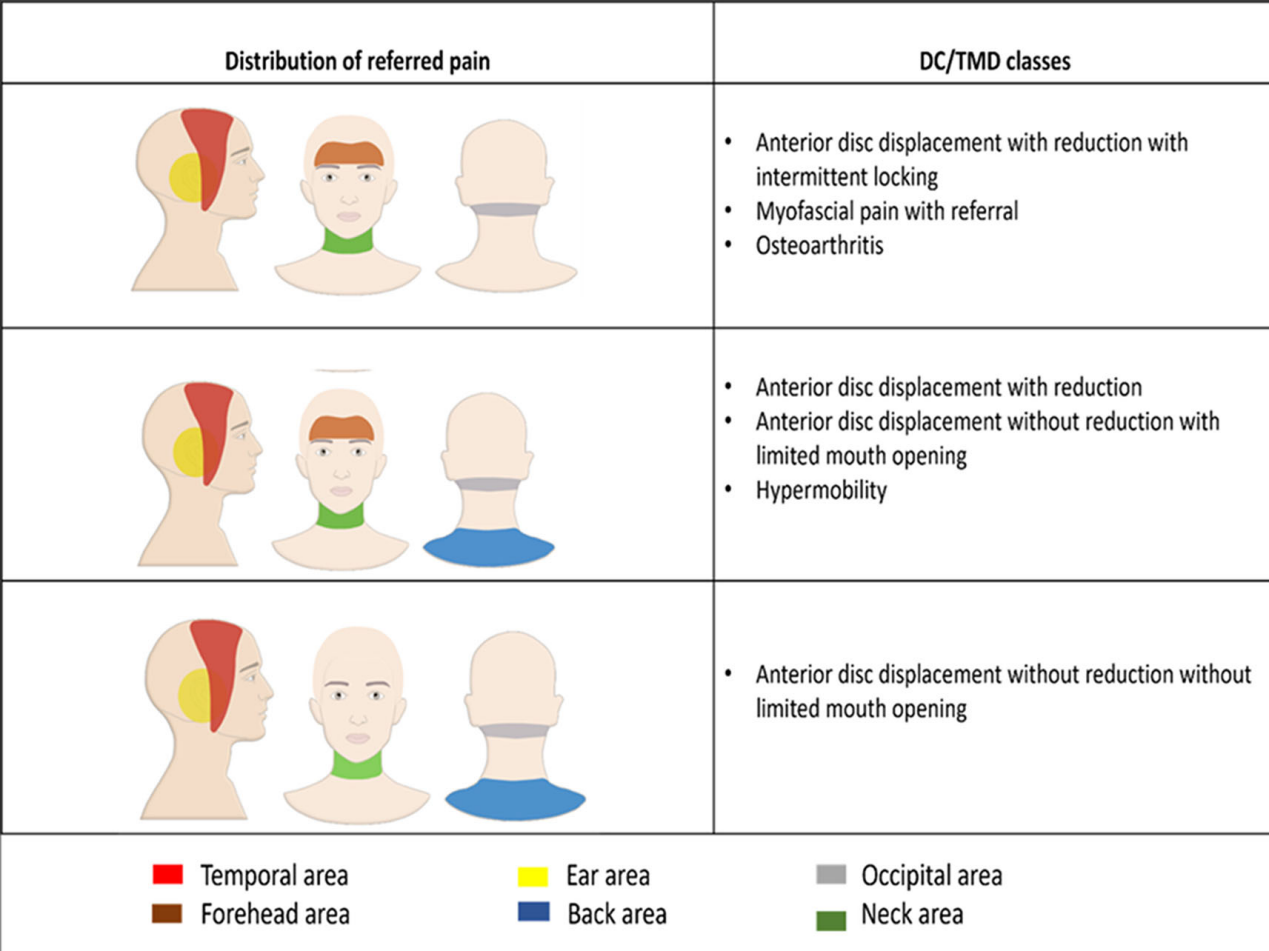
The referred pain distribution in different DC/TMD classes.

The referred pain was recorded in disc displacement with reduction with intermittent locking and myofascial pain with referral in all patients (100%), in osteoarthritis in 19 patients (90.48%) and in hypermobility in 11 patients (73.33%) ( Figure 2). The proportion of patients with referred pain was significantly different between the different DC/TMD diagnostic subgroups (P < 0.001). The Cramer’s V measure of association showed a strong strength association between the different diagnostic subgroups and the presence of referred pain (P < 0.001). Referred pain was recorded in anterior disc displacement with reduction in the temporal area in 35 patients (36.8%) and in the ear in 30 patients (31.6%). In anterior disc displacement without reduction with limited mouth opening, referred pain was recorded in the temporal in 20 patients (36.4%) and in the ear in 20 patients (36.4%). In myofascial pain with referral, referred pain was recorded in the temporal in 18 patients (29.0%) and in the ear in 18 patients (29.0%) ( Table 1 and Figure 3). The correlation between the different TMD classes and the referred pain locations was not significant (P > 0.05). The Cramer’s V measure of association showed a weak association between the different TMD classes and the referred pain locations (P > 0.05). The TMJ pain level recorded in patients with referred pain (5.29 ± 2.65) was significantly higher compared to the pain level recorded in patients without referred pain (4.12 ± 2.74) (P < 0.001). The Point-biserial correlation coefficient showed a weak strength association between the presence of referred pain and the TMJ pain level (P < 0.001). The TMJ pain duration in patients with referred pain (28.71 ± 39.10) was significantly higher than in patients without referred pain (21.66 ± 29.27) (P < 0.05). The Point-biserial Correlation Coefficient showed a very weak strength association between the presence of referred pain and the duration of TMJ pain (P > 0.05). The mouth opening in patients with referred pain (30.46 ± 9.56) was significantly lower than the mouth opening in patients without referred pain (41.24 ± 8.04) (P < 0.05). The Point-biserial Correlation Coefficient showed a weak strength association between the mouth opening and the presence of referred pain (P < 0.05). The disability score in patients with referred pain was significantly higher than in patients without referred pain (P < 0.001). The Rank biserial correlation coefficient showed a moderate strength association between the disability score and the referred pain (P < 0.001). The correlation between the masseter muscle and temporalis muscle tenderness and the presence of referred pain was significant (P < 0.001).

**Table 1.** Relation between the different DC/TMD classes and the referred pain locations.

		Temporal	Ear	Neck	Forehead	Occipital	Back	Total
**Diagnosis**	**Anterior disc displacement with reduction**	35	30	11	8	7	4	95
		36.8%	31.6%	11.6%	8.4%	7.4%	4.2%	100.0%
	**Disc displacement with reduction with intermittent locking**	2	3	1	1	1	0	8
		25.0%	37.5%	12.5%	12.5%	12.5%	0.0%	100.0%
	**Anterior disc displacement without reduction with limited mouth opening**	20	20	5	5	4	1	55
		36.4%	36.4%	9.1%	9.1%	7.3%	1.8%	100.0%
	**Anterior disc displacement without reduction without limited mouth opening**	5	5	4	0	1	2	17
		29.4%	29.4%	23.5%	0.0%	5.9%	11.8%	100.0%
	**Myofascial pain with referral**	18	18	11	12	3	0	62
		29.0%	29.0%	17.7%	19.4%	4.8%	0.0%	100.0%
	**Hypermobility**	6	6	2	5	2	2	23
		26.1%	26.1%	8.7%	21.7%	8.7%	8.7%	100.0%
	**Osteoarthritis**	19	19	6	10	1	0	55
		34.5%	34.5%	10.9%	18.2%	1.8%	0.0%	100.0%
**Total**		105	101	40	41	19	9	315
		33.33%	32.1%	12.7%	13%	6.03%	2.85%	100.0%

In anterior disc displacement with reduction, dull referred pain was reported in 41 patients (40.2%), sharp and throbbing referred pain was reported in 24 patients (23.5%) and heterogeneous referred pain was reported in 2 patients (1%). In anterior disc displacement with reduction with intermittent locking, dull referred pain was reported in 2 patients (66.7%) and sharp and throbbing referred pain was reported in 1 patient (33.3%) ( Table 2). The Cramer’s V Measure of Association showed a moderate strength association between pain character and different DC/TMD diagnoses (P < 0.001).

**Table 2.** Relation between the different DC/TMD classes and the referred pain character.

		Pain character				Total
		No	Dull	Sharp and throbbing	Heterogeneous	
**Diagnosis**	**Anterior disc displacement with reduction**	35	41	24	2	102
		34.3%	40.2%	23.5%	2.0%	100.0%
	**Anterior disc displacement with reduction with intermittent locking**	0	2	1	0	3
		0.0%	66.7%	33.3%	0.0%	100.0%
	**Anterior disc displacement without reduction with limited mouth opening**	1	11	22	0	34
		2.9%	32.4%	64.7%	0.0%	100.0%
	**Anterior disc displacement without reduction without limited mouth opening**	3	6	4	0	13
		23.1%	46.2%	30.8%	0.0%	100.0%
	**Myofascial pain with referral**	2	14	11	2	29
		6.9%	48.3%	37.9%	6.9%	100.0%
	**Hypermobility**	3	8	3	1	15
		20.0%	53.3%	20.0%	6.7%	100.0%
	**Osteoarthritis**	0	7	13	1	21
		0.0%	33.3%	61.9%	4.8%	100.0%
**Total**		44	89	78	6	217
		20.2%	41%	36%	2.8%	100.0%

**Figure 4.**
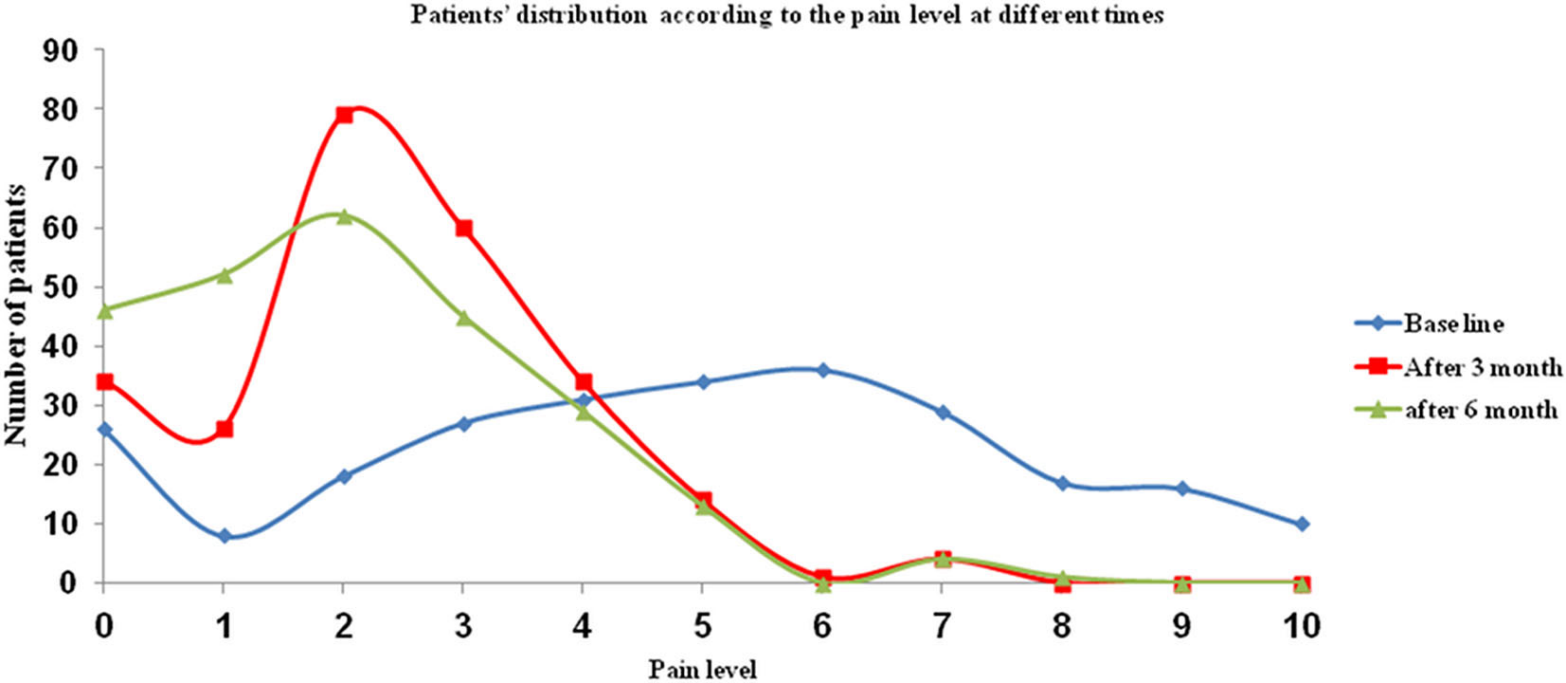
Patients’ distribution according to the referred pain levels at different times after treatment.

In the present study, 198 patients (78.6%) received the required treatment for their TMD. Following treatment, 83 patients (62.4%) reported improvement of their referred pain ( Figure 4). The referred pain level significantly decreased following treatment at 3 months (P < 0.001), and at 6 months (P < 0.001).

## Discussion

The referred pain often complicates the diagnosis of TMD and may lead to progression to advanced forms of the disorders before the proper diagnosis is reached. Referred pain is mainly due to the visceral and somatic senses that have been carried to the brain through the same neurons, accordingly patients are not capable to localize the origin of the pain. The convergence projection theory stated that the sensory information travel to the central nervous system (CNS) through nerves that converge to neurons. The number of neurons is less than the number of nerves so the information from multiple nerves has to converge to a less number of neurons to be carried to the CNS. The convergence appears at the level of the neurons that transmit impulses from deep structures like teeth pulp, joints, and muscles rather than from superficial structures. The neurons that originate from the TMJ and the masseter muscle appear mainly to converge in the trigeminal subnucleus caudalis. This theory elucidates the difficulty in localizing the pain which originates from the TMJ and the associated muscles and leads to referred pain away from the pain origin site. ^17^ The central sensitization theory also explains the referred pain mechanism through the continuous painful stimulus that can lead to the activation of distinct receptors that expand the sensitivity of neurons, thus widening the receptive field area. This changes the normal processing at the higher centers and causes nonpainful information to be perceived as painful. ^18^

In the present study, the prevalence of referred pain in patients diagnosed with TMD was 60.7%. Another study showed a lower prevalence of referred pain (26.4%), where the locations of referred pain were classified into extraoral, intraoral, and joint sites. According to this study, the TMD referred pain was diagnosed at the temporalis muscle (9.6%), masseter muscle (14.3%) and at the mandibular area (8.6%) whereas the joint area was involved in 7.5%. ^19^ The study was based on the RDC/TMD Validation and TMD Impact studies, whereas the present study was based on the modified DC/TMD classification that imposed new pain definitions and localization protocols. In the present study, the referred pain was recorded most commonly in the temporal area (45.2%) followed by the ear (42.1%) then the neck (19.0%). In another study, the TMD referred pain was reported most commonly in the cheek region followed by the ear and the forehead. The author of the study concluded that the pattern of TMD referred pain was consistent and predictable, which agrees to our study. ^17^ Another study reported the prevalence of referred TMD pain in the ear as 51.3%, which is in accordance with our results. ^20^

The evaluation of the prevalence of referred pain in different DC/TMD classes has shown that the most common location for referred pain in anterior disc displacement with reduction was in the temporal area (36.8%), in anterior disc displacement with reduction with intermittent locking was in the ear (37.5%), in anterior disc displacement without reduction with limited mouth opening was in the temporal area and the ear equally (36.4%) and in anterior disc displacement without reduction without limited mouth opening was in the temporal area and in the ear equally (29.4%). Referred pain was recorded in the ear and in the temporal area equally in hypermobility (26.1%), and in myofascial pain with referral (29.0%). These results are expected to facilitate the complex diagnostic processes of TMD by elaborating the possible pathways of referred pain related to each DC/TMD class.

In the present study, the association between referred pain and mouth opening was found to be weak. This result was not in accordance with another study that showed strong association between the mouth opening and referred pain in patients with TMD-related migraine. ^21^ There was a weak association in our study between temporalis muscle and masseter muscle tenderness and referred pain, and there was no association between lateral pterygoid muscle tenderness and referred pain. It has been shown that the presence of TMD-associated headaches in patients with myofascial pain does not negatively affect the treatment of pain; however it might change the pattern of pain improvement after the proper management. ^22^

The significant reduction in referred pain levels following different forms of treatment confirms that TMD was the cause of referred pain. Similar results were reported in another study that showed a reduction in the disability scores and referred pain levels following TMD treatment. ^21^

In the present study, the TMD referred pain characteristics were reported as dull pain in 42.5% of patients and as sharp and throbbing pain in 36.2% of patients. In another study, the TMD-related referred pain in the ear was described as severe and unbearable pain in 31.03% of patients, and as mild and bearable pain in 68.97% of patients. ^20^ The inconsistency in describing orofacial pain characteristics among various studies may negatively affect the scientific value of these studies. The authors of the present study recommend the unifying of the pain descriptive terms in different studies to maximize the benefit to the scientific community when comparing the results of these studies.

In conclusion, referred pain is a prominent feature of TMD. The prevalence of referred pain associated with TMD was 60.7%. The most common referred pain location was the temporal area, followed by the ear. Anterior disc displacement with reduction with intermittent locking and myofascial pain with referral were the most common diagnoses associated with referred pain. More studies are required with longer follow-up periods and bigger sample sizes to support the findings of the present study that are expected to significantly improve the diagnostic processes of TMD.

## Disclosures

This study is a part of a thesis submitted for partial fulfillment of the requirements of the Masters of Science Degree in Oral Surgery.

## Ethical approval

Research Ethics Committee, University of Sharjah.

## Consent

Written informed consent for publication of the participants/patients’ details and/or their images was obtained from the participants/patients/parents/guardian/relative of the participant/patient.

## Data availability

Repository: Harvard Dataverse: “Replication Data for: Prevalence and characteristics of referred pain in patients diagnosed with temporomandibular disorders according to the Diagnostic Criteria for Temporomandibular Disorders (DC/TMD)”, https://doi.org/10.7910/DVN/GXTCDR

This project contains the following underlying data:

• Data file 1. (Raw date used for assessment of the prevalence and evaluation of the characteristics of referred pain associated with temporomandibular disorders that are diagnosed according to the DC/TMD.)

Data are available under the terms of the Creative Commons Zero “No rights reserved” data waiver (CC0 1.0 Public domain dedication).
