# Supplementary material for: Prevalence and characteristics of referred pain in patients diagnosed with temporomandibular disorders according to the Diagnostic Criteria for Temporomandibular Disorders (DC/TMD) in Sharjah, United Arab Emirates
Source: F1000Res. 2022 Sep 12;11:656. Originally published 2022 Jun 14. [Version 2] doi: 10.12688/f1000research.109696.2 (PMC9490275; doi:10.12688/f1000research.109696.2)
Supplement: Peer review history

# Peer review: Reviewer response for version 2

**DOI:** 10.5256/f1000research.138218.r150101

**Published:** 2022-09-20

**Authors:** Khalifa Sulaiman Al-Khalifa

**Affiliations:** 1 Department of Preventive Dental Sciences, College of Dentistry, Imam Abdulrahman Bin Faisal University, Dammam, Saudi Arabia

All comments were addressed.

Is the work clearly and accurately presented and does it cite the current literature?

Partly

If applicable, is the statistical analysis and its interpretation appropriate?

Partly

Are all the source data underlying the results available to ensure full reproducibility?

Partly

Is the study design appropriate and is the work technically sound?

Partly

Are the conclusions drawn adequately supported by the results?

Partly

Are sufficient details of methods and analysis provided to allow replication by others?

Partly

Reviewer Expertise:

Dental Public Health

I confirm that I have read this submission and believe that I have an appropriate level of expertise to confirm that it is of an acceptable scientific standard.

# Peer review: Reviewer response for version 1

**DOI:** 10.5256/f1000research.121234.r140855

**Published:** 2022-08-17

**Authors:** Ahmed Ghoneima

**Affiliations:** 1 Department of Orthodontics, H Hamdan Bin Mohammed College of Dental Medicine, Mohammed Bin Rashid University of Medicine and Health Sciences, Dubai, United Arab Emirates

The submitted manuscript wishes to report the “Prevalence and characteristics of referred pain in patients diagnosed with temporomandibular disorders according to the Diagnostic Criteria for Temporomandibular Disorders (DC/TMD)”. The topic is of interest however at first reading some modifications maybe required to improve the quality of the manuscript. A proper rationale for the outlined hypothesis is not feasible without providing details of the demographic information of the included sample. In addition, authors should mention details of their inclusion and exclusion criteria. It will be surprising if the sample included for examples medically compromised patients who suffer from systematic diseases, on medications, previous surgery or TMJ problems, or diagnosed with bone diseases or osteoporosis. 

 The reviewer is also wondering whether the case diagnosis, evaluation, and data recording were done by only one investigator and whether the investigators had checked for intra- or inter-rater reliability. 

 It will also be very useful to the readers if the authors included the power of study analysis or sample size calculation.

Is the work clearly and accurately presented and does it cite the current literature?

Yes

If applicable, is the statistical analysis and its interpretation appropriate?

Yes

Are all the source data underlying the results available to ensure full reproducibility?

Yes

Is the study design appropriate and is the work technically sound?

Yes

Are the conclusions drawn adequately supported by the results?

Yes

Are sufficient details of methods and analysis provided to allow replication by others?

Yes

Reviewer Expertise:

Orthodontics, 3D imaging, Airway Analysis, Obstructive Sleep Apnea (OSA), Virtual Reality (VR), Augmented Reality (AR), and Artificial Intelligence (AI)

I confirm that I have read this submission and believe that I have an appropriate level of expertise to confirm that it is of an acceptable scientific standard.

# Response

**Published:** 2022-09-02

**Authors:** Wael Talaat

**Affiliations:** University of Sharjah, United Arab Emirates

**Response to Reviewer 2: Ahmed Ghoneima**

 The authors would like to express their gratitude for the reviewer for his constructive comments. We are hereby providing a point by point response.

 Comment 1:

 A proper rationale for the outlined hypothesis is not feasible without providing details of the demographic information of the included sample.

 Response:

 More demographic details were added in the results section.

 Comment 2:

 Authors should mention details of their inclusion and exclusion criteria.

 Response:

 The inclusion and exclusion criteria were elaborated in the Methods Section.

 Comment 3:

 The reviewer is also wondering whether the case diagnosis, evaluation, and data recording were done by only one investigator and whether the investigators had checked for intra- or inter-rater reliability. 

 Response:

 The TMJ clinical evaluation was carried out by two examiners; an oral and maxillofacial surgeon and a general dental practitioner who did the initial screening. The surgeon was blinded to the outcome of the initial screening procedure as well as to any related outcomes that were evaluated in all questionnaires. Calibration of the examiners based on the DC/TMD protocol was accomplished before the start of the study by asking each examiner to assess the same 20 subjects independently. The results were then analyzed, and any disagreements were resolved through group discussion. Inter-rater reliability was measured using the intraclass correlation coefficients (ICCs). The radiographic evaluations were analyzed twice, with a 1-week interval, by one specialist in oral radiology. The radiologist was blinded to the clinical evaluation outcomes. Intra-rater reliability was measured using the intraclass correlation coefficients (ICCs). Excellent inter-rater and intra-rater reliability was achieved for the clinical and radiographic diagnosis respectively (ICC=0.95-0.96).

 Comment 4:

 It will also be very useful to the readers if the authors included the power of study analysis or sample size calculation.

 Response:

 The formula used for the sample size calculation was added to the Methods Section.

# Peer review: Reviewer response for version 1

**DOI:** 10.5256/f1000research.121234.r140854

**Published:** 2022-07-19

**Authors:** Khalifa Sulaiman Al-Khalifa

**Affiliations:** 1 Department of Preventive Dental Sciences, College of Dentistry, Imam Abdulrahman Bin Faisal University, Dammam, Saudi Arabia

Title

- The study was conducted in Sharja, UAE - this needs to be reflected in the title and aim of your study.

Introduction

- No prevalence studies in the introduction.
- Explain Axis-I assessment algorithms for the reader.

Methods

- Where is the reference for Helkimos clinical dysfunction index?
- All instruments used - you need to use references. The author needs to cite all instruments used in the study.
- Are the tools used in the study validated?
- What is the gold standard for TMD?
- Spelling mistakes e.g., month vs mouth.
- Please arrange the diagnosis to be the same for figures and tables. 

Tables

- Table 1 - mention n(%) in one cell instead of two.
- Figure 3 is not clear, it would be better to remove the figure to avoid confusion.
- Combine table 1 and 2.

Discussion & Conclusion

- First part of the discussion should be in the introduction.
- You need to cite more papers in the discussion unless the information provided is new and has not been conducted in this case state that this clearly. 
- Limitations of the study should be stated before conclusion.
- Please re-write your conclusion based on your results.

Is the work clearly and accurately presented and does it cite the current literature?

Partly

If applicable, is the statistical analysis and its interpretation appropriate?

Partly

Are all the source data underlying the results available to ensure full reproducibility?

Partly

Is the study design appropriate and is the work technically sound?

Partly

Are the conclusions drawn adequately supported by the results?

Partly

Are sufficient details of methods and analysis provided to allow replication by others?

Partly

Reviewer Expertise:

Dental Public Health

I confirm that I have read this submission and believe that I have an appropriate level of expertise to confirm that it is of an acceptable scientific standard, however I have significant reservations, as outlined above.

# Response

**Published:** 2022-09-02

**Authors:** Wael Talaat

**Affiliations:** University of Sharjah, United Arab Emirates

**Response to Reviewer 1: Khalifa Sulaiman Al-Khalifa**

 The authors would like to express their gratitude for the reviewer for his constructive comments. We are hereby providing a point by point response.

 Comment 1:

 Title: The study was conducted in Sharjah, UAE - this needs to be reflected in the title and aim of your study.

 Response:

 It has been indicated in the title and aim that the study was conducted in Sharjah, United Arab Emirates.

 Comment 2:

 Introduction: No prevalence studies in the introduction.

 Response:

 The related prevalence studies were added in the introduction.

 Comment 3:

 Explain Axis-I assessment algorithms for the reader.

 Response:

 Axis-I assessment algorithms have been explained in the Introduction Section.

 Comment 4:

 Where is the reference for Helkimos clinical dysfunction index?

 Response:

 The reference was added in the Methods Section.

 Comment 5:

 All instruments used - you need to use references. The author needs to cite all instruments used in the study.

 Response:

 The missing citations were added in the Methods Section.

 Comment 6:

 Are the tools used in the study validated?

 Response:

 Yes, only validated tools were used in this study. An indication was added in the Methods Section.

 Comment 7:

 What is the gold standard for TMD?

 Response:

 The DC/TMD (Schiffman E, et al, 2014) is an objective and reliable diagnostic protocol that uses several validated assessment tools with high sensitivity and specificity. Magnetic resonance imaging is usually required to diagnose soft tissue disorders whereas cone beam computed tomography is needed to evaluate the bony changes.

 Comment 8:

 Spelling mistakes e.g., month vs mouth.

 Response:

 The spelling mistakes were corrected.

 Comment 9:

 Please arrange the diagnosis to be the same for figures and tables.

 Response:

 The diagnosis was arranged to be the same for figures and tables.

 Comment 10:

 Table 1 - mention n(%) in one cell instead of two.

 Response:

 Table 1 was modified and n(%) was mentioned in the same cell.

 Comment 11:

 Figure 3 is not clear, it would be better to remove the figure to avoid confusion.

 Response:

 Figure 3 was modified so that it is clearer to the reader.

 Comment 12:

 Combine table 1 and 2.

 Response:

 Tables 1 and 2 were combined in one table (table 1).

 Comment 13:

 First part of the discussion should be in the introduction.

 Response:

 The authors totally understand and value the comment of the reviewer. In the Introduction Section, the authors define the TMD referred pain as described by the DC/TMD and explain the reason why this type of pain often complicates the diagnosis of TMD. In the discussion section, the authors explain the neuroanatomic and physiologic theories of the referred pain.

 Comment 14:

 You need to cite more papers in the discussion unless the information provided is new and has not been conducted in this case state that this clearly. 

 Response:

 Although many studies evaluated the pain associated with the different classes of TMD, a limited number of studies were concerned with the TMD-associated referred pain. This was elaborated in the first paragraph in the Discussion Section.

 Comment 15:

 Limitations of the study should be stated before conclusion.

 Response:

 The limitations of the study were added before the conclusions.

 Comment 16:

 Please re-write your conclusion based on your results.

 Response:

 The conclusions were re-written based on the results.
